# Calculation of the Increase in Dose Rate Due to Precipitation at the Bilbao Radiological Station of the Basque Country Radiological Surveillance Network

**DOI:** 10.3390/s26144560

**Published:** 2026-07-18

**Authors:** Natalia Alegría, Miguel Ángel Hernández-Ceballos, Igor Peñalva, Fernando Legarda

**Affiliations:** 1 Energy Engineering Department, University of the Basque Country, 48013 Bilbao, Spain; igor.penalva@ehu.eus (I.P.); f.legarda@ehu.eus (F.L.); 2 Department of Physics, University of Cordoba, 14071 Cordoba, Spain; f92hecem@uco.es

**Keywords:** environmental monitoring, dose rate, radon progeny, precipitation, radiological surveillance, atmospheric washout

## Abstract

The Radiological Surveillance Network of the Autonomous Community of the Basque Country continuously monitors environmental dose rate levels to detect potential radiological anomalies. However, during rainfall events, increases in dose rate that occasionally exceed predefined alarm thresholds have been observed, despite the absence of radiological incidents. This study presents a predictive model to estimate the increase in dose rate caused by precipitation events at the Bilbao monitoring station. The model accounts for the atmospheric scavenging of radon progeny by raindrops and their subsequent deposition on horizontal surfaces surrounding the detector. Atmospheric transport, radioactive decay, washout processes, and rainwater accumulation dynamics are incorporated into the formulation. The model is implemented numerically using a finite-difference scheme and validated against experimental measurements from the monitoring station. The results (correlation coefficient, BIAS, RMSE) show good agreement between calculated and measured dose rate increases during precipitation episodes, indicating that the observed variations are mainly attributable to the deposition of short-lived radon progeny.

## 1. Introduction

Environmental radiological monitoring networks play a fundamental role in the early detection of abnormal increases in environmental radiation levels that may be associated with accidental or unauthorized releases of radioactive material [1,2]. These systems typically consist of distributed stations equipped with automatic detectors that continuously measure ambient gamma dose rate and transmit the data in real time to centralized control centers [3]. Such networks are widely implemented at national and regional scales as part of emergency preparedness and environmental protection programs [1,4,5,6].

Despite their effectiveness, dose rate monitoring systems are sensitive not only to artificial sources of radiation but also to variations produced by natural processes [7]. Among these processes, precipitation events are known to cause temporary increases in the environmental gamma dose rate. This phenomenon has been widely observed in monitoring stations located in different climatic regions and is generally attributed to the atmospheric washout of radionuclides belonging to the radon decay chain [8,9,10]. Radon (^222^Rn), a naturally occurring radioactive gas continuously emitted from soils and rocks, decays in the atmosphere, producing short-lived progeny such as ^214^Pb and ^214^Bi [7,11]. These radionuclides attach to atmospheric aerosols and can be efficiently scavenged by falling raindrops during precipitation events [9,12]. Once removed from the atmosphere, they are deposited on the ground and nearby surfaces, where their gamma emissions temporarily increase the ambient dose rate measured by monitoring detectors [8,10]. Because these radionuclides have short half-lives, the resulting dose rate enhancement is typically transient and closely linked to the timing and intensity of precipitation.

The interpretation of these precipitation-induced dose rate increases represents an important operational issue for radiological surveillance networks. In some cases, the magnitude of the increase can approach or exceed alarm thresholds established to detect potential radiological incidents [3,13,14,15]. Without a correct understanding of the underlying natural processes, these variations may generate false alarms or require unnecessary verification procedures by the monitoring authorities [13]. For this reason, several studies have investigated the relationship between rainfall intensity, atmospheric radon progeny concentrations, and the resulting variations in environmental dose rate [9,10,12]. Distinguishing these natural meteorological effects from radiological anomalies is therefore essential for the reliable interpretation of automatic monitoring data.

The Radiological Surveillance Network of the Autonomous Community of the Basque Country includes a set of automatic monitoring stations designed to continuously measure environmental radiation levels across the region. During the operation of the Bilbao station, repeated increases in dose rate were observed during precipitation episodes. These increases were not associated with artificial radioactive releases but were consistent with the deposition of short-lived radon progeny scavenged from the atmosphere by rainfall [8,9].

To quantitatively interpret these observations, a model is required to describe the physical processes governing the transfer of radionuclides from the atmosphere to the ground during precipitation events. Such processes include the atmospheric transport of radon progeny, their removal from the air by washout mechanisms, their incorporation into rainwater, the subsequent deposition on horizontal surfaces surrounding the detector, and the radioactive decay of the deposited radionuclides that produces the observed gamma radiation field [7,10]. A process-based model is particularly useful because it links the measured dose rate response to the sequence of atmospheric removal, deposition, accumulation, and radioactive decay processes.

The objective of this work is to develop and evaluate a numerical model that estimates the increase in environmental dose rate produced by precipitation events at the Bilbao radiological monitoring station. The model describes the atmospheric washout of radon progeny, their transport within the rainwater phase, and their accumulation and decay after deposition on surfaces near the detector. The resulting gamma radiation field is then converted into dose rate using conversion factors obtained through Monte Carlo simulations [16]. The model is implemented using a finite-difference numerical scheme, and its predictions are compared with dose rate measurements recorded during several precipitation episodes at the monitoring station.

The results obtained provide a quantitative explanation for the observed increases in environmental dose rate during rainfall events and demonstrate that they can be largely attributed to the deposition of short-lived radon progeny. This approach contributes to improving the interpretation of data from automatic radiological monitoring networks and helps reduce the occurrence of false alarms associated with natural atmospheric processes [8,13].

Previous studies [17,18,19,20,21,22,23,24] have mainly focused on observational analyses of dose rate increases during precipitation events or on the characterization of radon progeny scavenging processes. However, relatively few studies have developed operational models capable of quantitatively predicting the temporal evolution of dose rate increases at environmental monitoring stations. Therefore, there remains a need for process-based tools that can support real-time interpretation of radiological surveillance data and reduce false alarms associated with natural atmospheric processes.

## 2. Materials and Methods

### 2.1. Study Area

**Figure 1:**
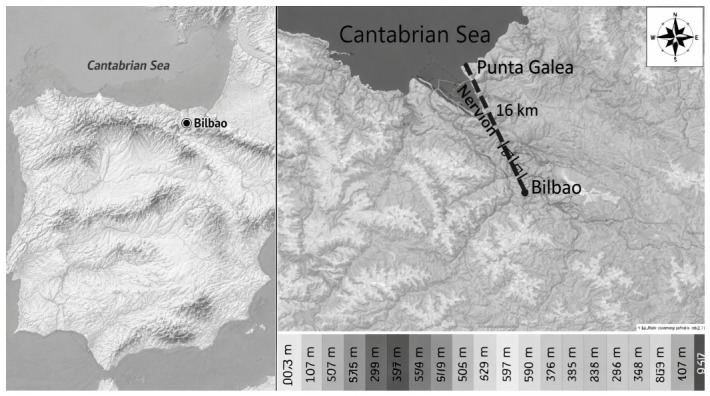
Location of Bilbao.

The study was conducted using data from the radiological monitoring station located in Bilbao, northern Spain. Bilbao (Figure 1) is situated on the northern coast of the Iberian Peninsula, within the Cantabrian climatic region, and is characterized by a humid oceanic climate with frequent precipitation throughout the year.

The average annual precipitation in the area is approximately 1200–1400 mm, with rainfall occurring on a large number of days each year. These meteorological conditions make the region particularly suitable for investigating the influence of precipitation on environmental radiation measurements. The monitoring station is installed in an urban environment within the metropolitan area of Bilbao. The city is located near the mouth of the Nervión River and is surrounded by mountainous terrain, with elevations typically ranging between 200 and 700 m above sea level [25,26]. The surrounding topography and proximity to the Bay of Biscay influence the local meteorology, frequently favouring low cloud bases and persistent stratiform precipitation systems. These characteristics are relevant to the present study because they affect the occurrence, duration, and vertical structure of rainfall events, which are key factors in the atmospheric scavenging of radon progeny.

### 2.2. Monitoring Equipment

**Figure 2:**
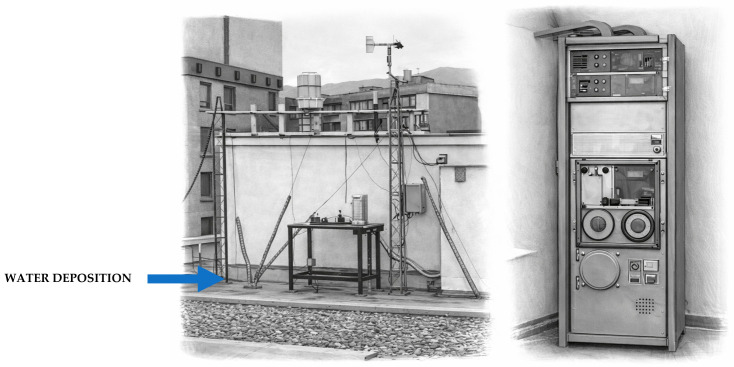
(**left side**) Meteorological station and (**right side**) radiological station of Bilbao.

The station operates continuously and records both radiological and meteorological variables required to analyze variations in environmental radiation levels during precipitation events (Figure 2). Ambient gamma dose rate is measured using a proportional counter detector specifically designed for environmental monitoring applications. The detector operates with an intrinsic background of approximately 1 count per second and a dead time of 1 μs. The sensitive volume of the detector consists of a cylindrical tube with a window diameter of 53 mm and a length of 508 mm. The instrument is designed to operate under a wide range of environmental conditions, with an operating temperature range from −30 °C to 60 °C and a relative humidity tolerance between 10% and 95%. These characteristics allow the detector to perform stable long-term measurements under outdoor conditions. The detector continuously measures the intensity of gamma radiation in the surrounding environment and converts the detected pulses into dose rate values. These measurements provide a direct indication of variations in the ambient radiation field, and they are used as the primary radiological variable in this study.

Precipitation data are obtained using a rain gauge installed at the monitoring site. The instrument has a collection area of 20,000 mm^2^, an external diameter of 180 mm, and a length of 30 mm. The rain gauge is designed to operate within a temperature range from −20 °C to 50 °C, allowing reliable measurements under typical meteorological conditions encountered in the region.

Both the radiological and meteorological sensors are connected to the station data acquisition system, which automatically records the measurements at regular time intervals of 10 min. This sampling interval provides sufficient temporal detail to analyze the evolution of radiation levels during precipitation events while maintaining manageable data volumes for long-term monitoring. All results are presented using a 10-min time resolution, corresponding to the acquisition interval of the monitoring system. These measurements constitute the experimental basis used in this work to analyze rainfall episodes and to validate the numerical model developed to estimate the increase in environmental dose rate associated with the atmospheric washout and deposition of radon progeny.

### 2.3. Model Description

The model developed in this work aims to estimate the increase in environmental dose rate recorded at the radiological monitoring station of Bilbao during precipitation events. The approach assumes that the observed increase is mainly caused by the atmospheric washout of short-lived radon progeny and their subsequent deposition on horizontal surfaces surrounding the detector. The model describes the sequence of processes that occur between the atmosphere and the detector environment during rainfall events, namely:

- The distribution and transport of radon and its progeny in the atmosphere;
- The removal of airborne radionuclides by precipitation (washout);
- The transport of radionuclides in the rainwater phase;
- The deposition and accumulation of activity on horizontal surfaces near the detector;
- The radioactive decay of deposited radionuclides and the generation of the gamma radiation field measured as dose rate.

These processes are represented using a set of balance equations describing radionuclide transport in the atmosphere, transfer to the rainwater phase, and deposition on surfaces. The resulting system is solved numerically using a finite difference scheme.

#### 2.3.1. Model Assumptions

The formulation is based on several assumptions concerning atmospheric conditions and the behaviour of radon progeny during precipitation events. The increase in dose rate is assumed to originate from the deposition of radon progeny present in the atmosphere onto surfaces located near the detector. These radionuclides are scavenged by falling raindrops before reaching the ground.

Meteorological observations indicate that approximately 80% of cloudy days in Bilbao are characterized by low cloud bases, typically associated with nimbostratus or stratus clouds. Under these conditions precipitation generally occurs as stratiform rainfall, which promotes efficient scavenging of atmospheric aerosols by raindrops. Raindrops are assumed to begin their descent from the cloud base. The contribution of radionuclides inside the cloud is neglected because their concentration is estimated to be approximately 10% of the concentration outside the cloud. Finally, the diameter of raindrops is assumed to depend on rainfall intensity, which in turn determines the terminal fall velocity of the droplets and therefore the downward transport velocity of radionuclides incorporated into rainwater [27].

**Figure 3:**
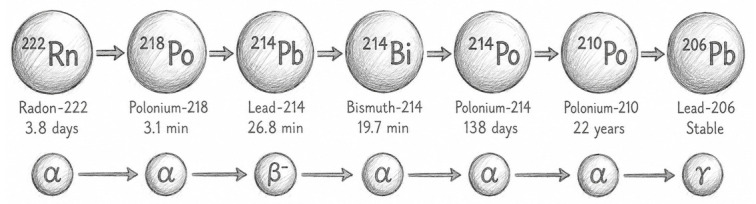
^220^Rn decay chain.

The isotope ^220^Rn (thoron) is not considered in the model due to its significantly lower atmospheric concentration compared with ^222^Rn. Consequently, the radionuclides included in the model correspond to the decay chain of ^222^Rn, particularly ^218^Po, ^214^Pb and ^214^Bi (see Figure 3).

Because the half-lives of ^214^Pb and ^214^Bi are relatively short (18.9 min and 26.9 min, respectively), rainfall events analyzed in the study must be separated by dry periods of at least 100 min in order to avoid interference between successive precipitation episodes.

#### 2.3.2. Atmospheric Compartment

To analyze the behaviour of radionuclides during precipitation, a representative atmospheric control volume above the monitoring station is defined. Within this control volume two phases coexist:

- an air phase, containing radionuclides attached to atmospheric aerosols;
- a water phase, present during rainfall and consisting of falling raindrops that scavenge atmospheric radionuclides.

**Figure 4:**
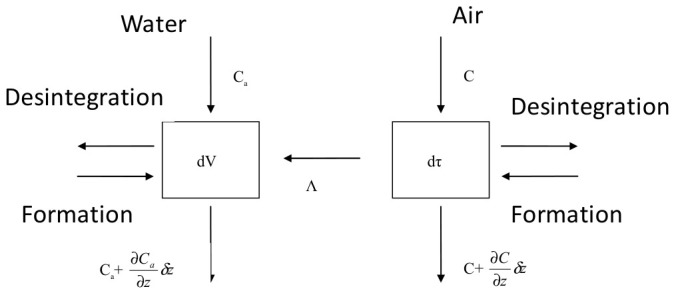
Scheme of the water and air phases.

The interaction between these phases is represented by the transfer of radionuclides from the air phase to the water phase through precipitation washout. The relationships between the different fluxes and control volumes are schematically represented in Figure 4.

The governing equation is based on the general atmospheric transport equation for radioactive aerosols, including advection, diffusion, radioactive decay, sedimentation and precipitation scavenging terms [28,29,30].

The concentration of radionuclide *i* in the air phase is described by the atmospheric transport equation

$$\frac{\delta C_{i}}{\delta_{t}}=\nabla \cdot \left(K\cdot \nabla C_{i}\right)-u\cdot \nabla C_{i}+v_{i}\cdot \frac{\delta C_{i}}{\delta_{z}}+\lambda_{i-1}\cdot C_{i-1}-\left(\lambda_{i}+\Delta_{i}\right)\cdot C_{i}$$

where:

*C*_i_ is the activity concentration of radionuclide iii in the air phase;
*K* is the coefficient of turbulent diffusion;
*u* is the average wind velocity;
*v*_i_ is the sedimentation velocity of radionuclide *i*;
*λ*_i_ is the radioactive decay constant of radionuclide *i*;
$\Delta_{i}$ is the washout coefficient describing the removal of radionuclides by precipitation.

In the present study several simplifications are introduced to focus on the dominant processes. Turbulent diffusion terms are neglected because they are at least two orders of magnitude smaller than the dominant terms. The vertical component of the wind velocity is also neglected, and the sedimentation term is considered negligible compared with the radioactive decay term. Under these assumptions the evolution of radionuclide concentration is mainly governed by radioactive decay and precipitation washout.

#### 2.3.3. Water Phase

Radionuclides removed from the atmosphere are incorporated into falling raindrops and transported toward the ground in the rainwater phase. The activity balance for radionuclides transported by precipitation can be expressed as

$$\frac{\delta A_{i}}{\delta_{t}}=-\lambda_{i}\cdot A_{i}+\lambda_{i}\cdot Q_{i}+\lambda_{i}\cdot A_{i-1}-D$$

where:

$A_{i}$ is the activity of the radionuclide *i* deposited in the roof for surface unit;
$\lambda_{i}$ is the radioactive decay constant of radionuclide i;
$Q_{i}$ is the number of nuclei deposited per unit of time and by surface unit contributed by the rain;
D is the activity transported by the spilt one per unit of time and of surface.

##### Deposition on Horizontal Surfaces

When raindrops reach the ground, they break and spread over nearby horizontal surfaces. The activity of deposited radionuclides per unit surface area is calculated considering both deposition and radioactive decay:

$$\frac{\delta A_{i}}{\delta_{t}}=N-\lambda_{i}\cdot A_{i}$$

where *A*i is the deposited activity per unit area, and *Ni* is the deposition rate.

The set of equations describing the evolution of A radionuclide concentrations in the different phases is solved numerically using the finite difference method. The independent variables of the numerical scheme are height and time. Spatial steps are on the order of metres, while time steps are on the order of seconds, which is consistent with the range of precipitation intensities observed at the monitoring station.

##### Rainwater Accumulation and Drainage

After deposition, rainwater accumulates on the ground and surrounding surfaces. Due to surface tension effects, the accumulated water forms a thin layer whose thickness depends on the contact angle between the water and the surface.

Field observations indicate that the thickness of this water layer typically ranges between 2.5 mm and 3.5 mm for irregular surfaces containing impurities, with an average value of approximately 3 mm. When this level is exceeded, excess water is evacuated through drainage systems located on the detector roof.

The relationship between the accumulated water height and the evacuation flow is obtained using basic principles of fluid dynamics, namely the conservation of mass (continuity equation) and the conservation of energy expressed by Bernoulli’s equation.

#### 2.3.4. Dose Rate Calculation

The environmental dose rate produced by the deposited radionuclides is calculated as

$$\dot{D}=\frac{1}{T}\int A\cdot FCD\cdot dt=\frac{FCD}{T}\int A\cdot dt$$

Being *T* the duration of the interval in seconds, to the *A* activity and *FCD* the conversion to dose factor.

The factor FCD was calculated as the product of four terms: (i) the particle flux resulting from the decay of each radionuclide deposited on the horizontal surfaces surrounding the detector, calculated using the MCNP Monte Carlo code; (ii) the flux-to-air-kerma conversion factor; (iii) the effective-dose conversion factor, assuming ISO irradiation geometry, defined as a radiation field in which the particle fluence per unit solid angle is independent of direction; and (iv) the emission probability of the corresponding radiation.

### 2.4. Evaluation of Model Predictions

The relationship between variables was evaluated using the Pearson correlation coefficient (*r*), the root mean square error *(RMSE*) and the mean bias error (BIAS). The Pearson coefficient quantifies the strength and direction of the linear relationship between two variables and is defined as

$$r=\frac{\sum_{i=1}^{N}\left(x_{i}-\bar{x}\right)\cdot \left(y_{i}-\bar{y}\right)}{\sqrt{\sum_{i=1}^{N}{\left(x_{i}-\bar{x}\right)}^{2}\cdot \sum_{i=1}^{N}{\left(y_{i}-\bar{y}\right)}^{2}\cdot }}$$

It ranges between −1 and +1, where values close to +1 indicate a strong positive linear correlation, values close to −1 indicate a strong negative linear correlation, and values near 0 suggest the absence of a linear relationship.

The root mean square error (*RMSE*) provides information about the performance of a model by allowing a term-by-term comparison of the actual difference between the predicted and he measured value. This statistical index varies from 0 to +∞, and hence, the smaller the value of RMSE, the better the model’s performance.

$$RMSE=\sqrt{\frac{1}{N}\overset{n}{\underset{i=1}{\sum }}Q_{s,i}-Q_{0,i}^{2}}$$

where $Q_{s,i}$ denotes the simulated value and $Q_{o,i}$ denotes the corresponding observed value for the i-th data point.

In addition, the mean bias error (BIAS) was also used to evaluate the systematic deviation between the model predictions and the experimental measurements. BIAS is defined as the average difference between the predicted and observed values:

$$BIAS=\;\frac{1}{n}\overset{n}{\underset{i=1}{\sum }}\left(Q_{S,i}-Q_{O,i}\right)$$

where *Q*_*s*,*i*_ and *Q*_*o*,*i*_ are the observed and predicted values, respectively, and n is the number of data points. A BIAS value close to zero indicates the absence of systematic deviation between the model and the measurements. Positive values indicate that the model tends to overestimate the observed dose rate increase, whereas negative values indicate a tendency to underestimate it.

## 3. Results

The predictive capability of the proposed model was evaluated by comparing the simulated environmental dose-rate increases with the corresponding measurements recorded at the Bilbao radiological monitoring station during multiple independent precipitation episodes. The selected events covered different rainfall durations, precipitation intensities, and cloud-base heights, allowing the model performance to be assessed under a range of meteorological conditions. The analysis focused on rainfall episodes composed of consecutive precipitation intervals, as these conditions favour the progressive washout, deposition, and accumulation of radon progeny in the vicinity of the detector, leading to measurable dose-rate enhancements. Representative cases consisting of three, four, five, and six consecutive precipitation intervals were selected to illustrate the model behaviour and predictive capability under different rainfall scenarios. For each event, independent performance metrics, including the correlation coefficient, RMSE, and BIAS, were calculated to quantify the agreement between simulated and observed dose-rate increases. Although these representative cases demonstrate the ability of the model to reproduce precipitation-induced radiological responses, the present study should be regarded as a proof-of-concept validation, and future work will extend the analysis to a substantially larger database of precipitation events to further assess model robustness and generalizability.

A total of 50 independent precipitation events fulfilling the selection criteria were analyzed during this study. The representative cases presented below were selected because they illustrate the different rainfall durations and meteorological conditions considered. The remaining events showed similar model behaviour, with comparable agreement between simulated and measured dose-rate increases and did not reveal additional response patterns beyond those discussed in the representative examples.

The events not included in the figures exhibited comparable temporal evolution and similar statistical performance. Therefore, they were omitted for brevity, as their inclusion would not provide additional information regarding the behaviour of the proposed model.

**Figure 5:**
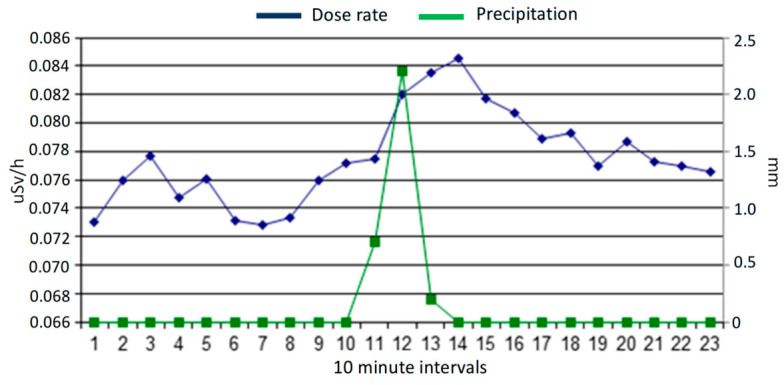
Example of the 10-min evolution of dose rate and precipitation.

To analyze dose rate behaviour, at least ten consecutive 10-min intervals without recorded precipitation are required before rainfall begins, ensuring that the influence of any previous rain event is negligible. This 100-min period allows the decay products of radon, specifically ^214^Pb and ^214^Bi deposited on the ground, to decay to insignificant levels due to their respective half-lives, while atmospheric concentrations are also expected to recover within approximately two hours. Similarly, after the rainfall event, another ten consecutive intervals without precipitation are required to examine the relaxation of the dose rate increase without interference. These criteria are defined in Figure 5.

### 3.1. Case with Three Precipitation Intervals

**Figure 6:**
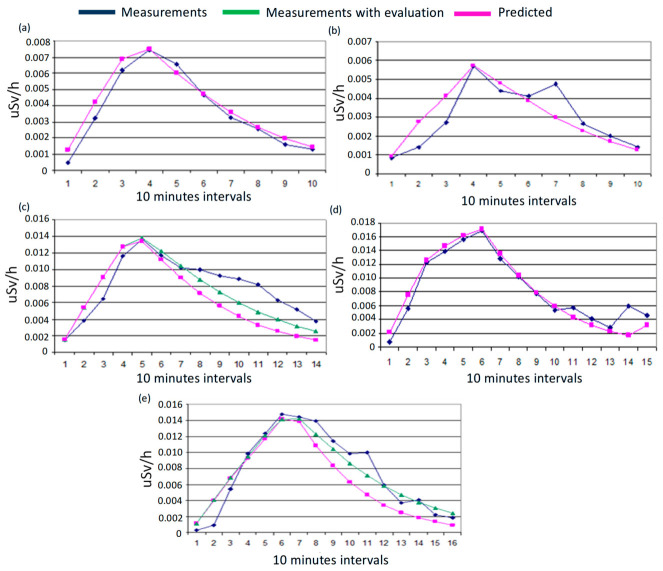
Temporal evolution (in 10 min intervals) of measurements and predictions under different episodes of precipitation intervals in (**a**) three, (**b**) three, (**c**) four, (**d**) five, (**e**) six precipitation intervals, respectively.

These events represent the shortest precipitation sequences capable of producing measurable increases in environmental dose rate. The first case corresponds to a rainfall episode with three precipitation intervals and rainfall amounts of 0.7, 2.2 and 0.2 L m^−2^. The effective cloud base height measured by the ceilometer was 304.8 ± 30.5 m, while a value of 300 m was used in the simulation. The drainage system of the detector roof was assumed to operate normally. The comparison between measured and calculated dose rate increases is presented in Figure 6a, where the measured values are represented by the blue curve and the model results by the red curve. The standard simulation (red curve), which assumes normal drainage conditions, is compared with an alternative simulation (green curve) assuming no drainage due to temporary obstruction.

The temporal evolution of the calculated dose rate follows closely the behaviour of the measured data during both the rising and descending phases of the event. Although the model assumes that rainfall occurs continuously throughout each 10-min interval, which may slightly modify the slope of the calculated curve, the overall trend is well reproduced. Both curves reach their maximum during the precipitation period and subsequently decrease as the deposited radionuclides decay.

A quantitative comparison between the measured and simulated values yielded a correlation coefficient of 0.99, indicating a very strong positive linear relationship between both series. The RMSE was 0.00025 µSv h^−1^, reflecting a very small deviation between the calculated and measured dose rate increases. In addition, the BIAS was −0.00003 µSv h^−1^, indicating an almost negligible systematic deviation and a very slight underestimation by the model.

A second event composed of three precipitation intervals was also analyzed. In this case the rainfall amounts were 0.7, 0.2 and 0.1 L m^−2^. The effective cloud base height measured during the event was 487 ± 49 m, and a value of 500 m was adopted in the simulation. Normal drainage conditions were assumed. The comparison between measured and calculated dose rate increases is shown in Figure 6b.

As observed in the previous case, the model reproduces the general evolution of the measured dose rate. Differences between measured and simulated values are mainly observed during the ascending phase of the event, which is related to the assumption of uniform rainfall within each 10-min interval in the model formulation. An isolated anomalous point appears in the measured series during interval 7. This value is interpreted as a statistical fluctuation rather than the effect of additional precipitation, since the subsequent decrease in dose rate follows the decay pattern predicted by the model. For this event, the calculated correlation coefficient was 0.6981, indicating a moderate positive relationship between the modelled and observed dose rate increases. The RMSE was 0.001201 µSv h^−1^, reflecting larger deviations than those obtained for the previous event but still within a limited range, while the BIAS was −0.000085 µSv h^−1^, indicating a slight tendency of the model to underestimate the measured values.

### 3.2. Case with Four Precipitation Intervals

The representative example considered here includes rainfall amounts of 0.5, 0.2, 0.9 and 0.4 L m^−2^. The effective cloud base height was 304.8 ± 30.5 m and a value of 300 m was used in the simulation. The comparison between measured and calculated dose rate increases is shown in Figure 6c. The standard simulation (red curve), which assumes normal drainage conditions, is compared with an alternative simulation (green curve) assuming no drainage due to temporary obstruction. During the ascending phase of the event the modelled values increase more rapidly than the measured values. This behaviour arises from the assumption that precipitation occurs continuously throughout each measurement interval, which causes radionuclides transported by rainfall to begin contributing to the radiation field immediately in the simulation.

In contrast, during the descending phase the calculated values assuming normal drainage are lower than the measured ones. To investigate this discrepancy, an additional simulation was performed assuming that water evacuation through the drainage system did not occur due to a temporary obstruction. The results of this alternative simulation are also presented in Figure 6c. Under this assumption, the agreement between modelled and observed values improves significantly. However, a small isolated increase observed later in the measured series suggests the occurrence of a very weak precipitation event that was not detected by the rain gauge due to its sensitivity threshold. Examination of the precipitation records indicates the presence of a rainfall value of 0.1 mm during a later interval, which supports the hypothesis of an unrecorded drizzle event.

For this event, the statistical comparison showed differences between the standard and alternative simulations. In the standard simulation, the correlation coefficient was r = 0.8954, the RMSE was 0.001819 µSv h^−1^, and the BIAS was +0.000608 µSv h^−1^, while in the alternative simulation, the correlation coefficient decreased to r = 0.8198, and the RMSE increased to 0.002772 µSv h^−1^ and the BIAS increased to +0.001549 µSv h^−1^. These results indicate that the standard simulation provided a better agreement with the measured values, as reflected by the higher correlation coefficient and the lower RMSE. The positive BIAS observed in both cases indicates a tendency of the model to overestimate the measured dose rate increase, although this overestimation was more pronounced in the smoothed simulation.

### 3.3. Case with Five Precipitation Intervals

This case corresponds to a rainfall event with five consecutive precipitation intervals, with recorded rainfall amounts of 0.8, 0.4, 0.2, 0.1, and 0.1 L m^−2^ (Figure 6d). The standard simulation (red curve), which assumes normal drainage conditions, is compared with an alternative simulation (green curve) assuming no drainage due to temporary obstruction. The effective cloud base height, measured by the ceilometer, was 304.8 ± 30.5 m, and the simulated cloud base height used in the model was 300 m. The drainage system of the detector roof was assumed to operate normally.

During this event, both the measured and calculated curves exhibited a similar temporal evolution. The dose rate increased gradually as precipitation continued and began to decrease after the rainfall intensity diminished. The general trends of both curves were closely aligned, demonstrating that the model effectively captured the main physical processes governing the increase in environmental dose rate. However, toward the end of the event, the model underestimated the measured values and did not reproduce a small increase observed in the measurements.

A correlation coefficient of 0.96 was obtained for this event, indicating a very strong positive linear relationship between the observed and calculated values. The BIAS was approximately −0.0165 µSv h^−1^, suggesting a systematic underestimation of the dose rate increase by the model. The RMSE was approximately 0.0280 µSv h^−1^, indicating a higher level of deviation between the modelled and observed dose rate increases than in the previous events.

### 3.4. Case with Six Precipitation Intervals

The longest precipitation sequence analyzed corresponds to an event consisting of six consecutive rainfall intervals with precipitation amounts of 0.3, 0.2, 0.7, 0.5, 0.1 and 0.1 L m^−2^. The effective cloud base height measured during the event was 274.3 ± 27.4, while a simulated value of 300 m was adopted. The comparison between measured and calculated dose rate increases is presented in Figure 6e. The standard simulation (red curve), which assumes normal drainage conditions, is compared with an alternative simulation (green curve) assuming no drainage due to temporary obstruction. During the ascending phase of the precipitation episode, particularly between intervals 4 and 6, the model reproduces the measured increase in dose rate with good accuracy. However, during the descending phase the measured values remain higher than those calculated when drainage is considered.

As in the previous case, an additional simulation assuming the absence of drainage was performed. The results of this simulation are presented in Figure 6e, where the calculated values show a closer agreement with the measured data. The remaining differences are attributed to minor precipitation contributions that were not recorded by the rain gauge due to their small magnitude. For this event, the statistical comparison showed clear differences between the standard and modified simulations. In the standard simulation, the Pearson correlation coefficient was r = 0.915, the BIAS was −0.00123 µSv h^−1^, and the RMSE was 0.00233 µSv h^−1^, while in the modified simulation, the correlation coefficient increased to r = 0.972, while the BIAS decreased to −0.000038 µSv h^−1^ and the RMSE decreased to 0.00133 µSv h^−1^. These results indicate that the modified simulation provided a better agreement with the measured values, as reflected by the higher correlation coefficient and the lower RMSE. The negative BIAS observed in both cases indicates a slight tendency of the model to underestimate the measured dose rate increase; however, this underestimation was almost negligible in the modified simulation.

## 4. Discussion

The results obtained in this study indicate that the proposed model is capable of reproducing the temporal evolution of environmental dose-rate increases associated with precipitation events at the Bilbao radiological monitoring station. Overall, the model successfully captures the magnitude and timing of the observed responses, although its performance varies among events, with some episodes exhibiting lower correlation coefficients and larger prediction errors. The generally high correlation coefficients and low RMSE values obtained for most of the analyzed events suggest that the model adequately represents the dominant physical mechanisms governing the washout, deposition, accumulation, and radioactive decay of radon progeny during rainfall episodes [6,7,8]. Furthermore, the BIAS values were generally close to zero, indicating the absence of a significant systematic tendency towards either overestimation or underestimation of the observed dose-rate increases. Nevertheless, negative BIAS values observed in some cases indicate a slight underprediction of the measured response, particularly during the later stages of certain precipitation events.

The simulated temporal patterns are consistent with the physical behaviour reported in previous investigations of precipitation-induced dose-rate enhancements. Numerous studies have demonstrated that rainfall-driven scavenging of radon progeny from the atmosphere, followed by their deposition on the ground surface, constitutes the primary mechanism responsible for short-term increases in environmental gamma dose rate [8,9,10]. The characteristic increase during precipitation and subsequent decrease governed by radioactive decay processes observed in the present work is therefore consistent with the behaviour reported in previous experimental and modelling studies [8,9,10]. Moreover, the magnitude of the simulated dose-rate increases is comparable to values reported in the literature [8], supporting the assumption that short-lived radionuclides, particularly ^214^Pb and ^214^Bi, represent the dominant contributors to the observed radiological response.

Despite the overall agreement between simulations and observations, several sources of uncertainty may affect model performance. One important limitation arises from the assumption that precipitation intensity remains constant within each 10-min measurement interval. In reality, rainfall intensity may exhibit substantial intra-interval variability, leading to temporal fluctuations in scavenging efficiency that are not explicitly represented in the current model formulation. Consequently, the assumption of uniform rainfall may introduce discrepancies between simulated and measured dose-rate responses, particularly during rapidly evolving precipitation events. Similar limitations have been identified in previous studies, where short-term variability in precipitation characteristics was shown to significantly influence radionuclide washout processes [9].

Additional uncertainty is associated with the simplified representation of atmospheric scavenging mechanisms. Previous studies have demonstrated that washout efficiency depends on several meteorological parameters, including rainfall intensity, droplet size distribution, atmospheric stability, and cloud microphysical properties [8,10]. In the present formulation, these complex processes are represented through a constant washout coefficient, which provides a practical simplification but may not fully capture event-to-event variability. This simplification is likely to contribute to some of the discrepancies observed during the onset of precipitation, when scavenging processes are particularly sensitive to changing meteorological conditions [6,10]. Future developments could incorporate dynamic washout parameterizations linked to rainfall characteristics in order to improve predictive capability.

Another source of uncertainty is related to precipitation measurements. The rain gauge employed in this study is subject to a minimum detection threshold, which may prevent the identification of very light precipitation events. Although such events contribute only limited amounts of rainfall, they may still produce measurable deposition of radon progeny and consequently generate detectable increases in environmental dose rate. As a result, some low-intensity radiological responses may not be fully reproduced by the model. Similar limitations associated with precipitation measurement thresholds have been reported previously [10]. This limitation highlights the importance of improving precipitation monitoring capabilities and exploring complementary measurement techniques capable of characterizing weak precipitation events more accurately.

The analysis also revealed differences in model performance among the investigated precipitation episodes. In particular, the event composed of five consecutive precipitation intervals exhibited larger BIAS and RMSE values than the remaining cases. This behaviour suggests that additional factors not explicitly represented in the current formulation may influence radionuclide deposition and retention under certain meteorological conditions. Possible contributors include uncertainties in cloud-base height estimation, spatial variability in rainfall intensity, local drainage conditions, and surface retention processes near the detector. Consequently, while the model reproduces the overall behaviour of most analyzed events, further validation using a larger and more diverse dataset is required to fully assess its robustness and general applicability.

It should also be noted that the present study constitutes a retrospective validation exercise based on selected precipitation episodes. The measured dose-rate data were used exclusively for model evaluation and performance assessment, whereas the model predictions were generated from independent meteorological and physical parameters. Therefore, the results should be interpreted as a proof-of-concept demonstrating the capability of the proposed framework to reproduce precipitation-induced dose-rate increases rather than as a comprehensive operational validation.

From an operational perspective, the model has potential applications as a decision-support tool within environmental radiological monitoring networks. By providing an estimate of the expected natural contribution to dose-rate increases during precipitation events, the model could assist operators in distinguishing between meteorologically induced fluctuations and anomalies requiring further investigation. However, practical implementation would require additional validation under a broader range of meteorological conditions and the incorporation of site-specific factors, including drainage characteristics and local environmental conditions. In a real-time deployment, such parameters would need to be treated as independent operational inputs rather than being inferred retrospectively from model performance.

Future work should focus on extending the validation database to include a substantially larger number of precipitation events covering different seasons, rainfall regimes, and atmospheric conditions. Such analyses would enable a more rigorous assessment of model uncertainty, sensitivity, and transferability. In addition, the incorporation of more detailed meteorological information, including variable washout coefficients, rainfall microphysics, and improved precipitation measurements, could further enhance model performance and support its eventual implementation in operational radiological surveillance systems.

## 5. Conclusions

This study presents a numerical model that successfully simulates the increase in environmental dose rate during precipitation events at the Bilbao radiological monitoring station. The model attributes these increases to the atmospheric washout and subsequent deposition of short-lived radon progeny. The comparison between modelled and measured dose rate increases shows good agreement, with high correlation coefficients and relatively low BIAS and RMSE values, indicating that the model can reliably predict the variations in dose rate during rainfall events.

While the model provides a robust explanation for the observed increases in environmental dose rate, some limitations exist. The assumption of uniform precipitation over each 10-min interval, along with the use of a constant washout coefficient, may introduce slight discrepancies between model predictions and actual measurements. Additionally, the sensitivity of the rain gauge and the potential influence of drainage issues on the deposition of radon progeny can impact the accuracy of the model’s predictions. Future improvements to the model could focus on incorporating more accurate precipitation data, variable washout coefficients, and more refined drainage system simulations.

Despite these limitations, the model proves to be a valuable tool for interpreting dose rate data during rainfall events. It provides an accurate and quantitative estimate of the contribution of radon progeny washout to the observed dose rate increase, which is crucial for distinguishing between natural radiation fluctuations and potential radiological incidents. The model’s ability to reduce false alarms and optimize monitoring protocols makes it an important addition to radiological surveillance networks.

In practical terms, the model can be integrated into real-time monitoring systems to provide automated predictions of dose rate increases during precipitation events, improving the efficiency of data interpretation and the speed of response to potential radiological anomalies. Furthermore, with adaptations to account for regional differences in meteorological conditions and washout characteristics, the model could be applied to a wide range of geographical regions, enhancing the accuracy and effectiveness of global radiological monitoring efforts.

Although the present study has been conducted as a retrospective validation exercise, the proposed modelling framework has been conceived with the objective of supporting environmental radiological surveillance systems in real-time operation. By estimating the expected contribution of precipitation-induced washout of radon progeny to environmental dose-rate increases, the model could assist operators in distinguishing natural atmospheric effects from potential radiological anomalies, thereby contributing to a more efficient interpretation of monitoring data.

Nevertheless, the current validation is based on a limited number of representative precipitation events and should therefore be regarded as a proof-of-concept rather than a comprehensive operational assessment. Additional validation using a substantially larger dataset covering different seasons, rainfall regimes, and meteorological conditions will be required to evaluate model robustness, uncertainty, and transferability. Furthermore, future work should address the integration of site-specific operational parameters and the implementation of the model within real-time radiological monitoring systems.

## Data Availability

The datasets used in this study are not publicly available because they belong to the Basque Country Radiological Surveillance Network. Data are available from the corresponding author upon reasonable request and subject to authorization by the data owner.
